# GPCRtm: An amino acid substitution matrix for the transmembrane region of class A G Protein-Coupled Receptors

**DOI:** 10.1186/s12859-015-0639-4

**Published:** 2015-07-02

**Authors:** Santiago Rios, Marta F. Fernandez, Gianluigi Caltabiano, Mercedes Campillo, Leonardo Pardo, Angel Gonzalez

**Affiliations:** Laboratori de Medicina Computacional, Unitat de Bioestadística, Facultat de Medicina, Universitat Autònoma de Barcelona, 08193 Bellaterra, Barcelona, Spain

**Keywords:** Amino acid substitution matrix, G protein-coupled receptors, GPCR, Transmembrane, Evolution, Membrane protein

## Abstract

**Background:**

Protein sequence alignments and database search methods use standard scoring matrices calculated from amino acid substitution frequencies in general sets of proteins. These general-purpose matrices are not optimal to align accurately sequences with marked compositional biases, such as hydrophobic transmembrane regions found in membrane proteins. In this work, an amino acid substitution matrix (GPCRtm) is calculated for the membrane spanning segments of the G protein-coupled receptor (GPCR) rhodopsin family; one of the largest transmembrane protein family in humans with great importance in health and disease.

**Results:**

The GPCRtm matrix reveals the amino acid compositional bias distinctive of the GPCR rhodopsin family and differs from other standard substitution matrices. These membrane receptors, as expected, are characterized by a high content of hydrophobic residues with regard to globular proteins. On the other hand, the presence of polar and charged residues is higher than in average membrane proteins, displaying high frequencies of replacement within themselves.

**Conclusions:**

Analysis of amino acid frequencies and values obtained from the GPCRtm matrix reveals patterns of residue replacements different from other standard substitution matrices. GPCRs prioritize the reactivity properties of the amino acids over their bulkiness in the transmembrane regions. A distinctive role is that charged and polar residues seem to evolve at different rates than other amino acids. This observation is related to the role of the transmembrane bundle in the binding of ligands, that in many cases involve electrostatic and hydrogen bond interactions. This new matrix can be useful in database search and for the construction of more accurate sequence alignments of GPCRs.

**Electronic supplementary material:**

The online version of this article (doi:10.1186/s12859-015-0639-4) contains supplementary material, which is available to authorized users.

## Background

G protein-coupled receptors (GPCRs) constitute a large family of integral membrane proteins that mediate numerous signaling pathways through second messenger cascades [1]. These receptors are activated by a vast chemical diversity of ligands, ranging from small molecules to lipids, peptides, or hormones [2] and display a highly conserved molecular architecture characterized by the presence of seven α-helical transmembrane segments (7TM) [3]. GPCRs are classified into six main families or classes (named A to F) based on sequence similarity, with only four of them (A, B, C and F) present in vertebrates [4]. The class A, also known as rhodopsin family [5], is the largest (~847 genes in humans) and exhibit a distinctive feature that most effector molecules bind to a cavity formed by the TM helices. The rhodopsin family is the subject of numerous studies due to their pharmacological relevance, representing the largest family of individual drug targets [6, 7].

The importance of the GPCRs in cellular physiology has inspired the development of numerous computational tools and databases for their study over the years [8–15]. The majority of these approaches have required multiple sequence alignments with very low identities (~20 %), in many cases below the twilight region significant for homology detection [16]. One important part of sequence alignment algorithms is the use of substitution matrices to account for the exchange rates of the amino acids within proteins [17]. Amino acid substitution matrices are obtained by the application of statistical methods on sequence alignments of evolutionarily related proteins (generally globular) and in all cases are biased by the composition of the data set used [18]. In this regard, it is known that the evolutionary selective pressure that governs the conservation and relative mutability of amino acids varies among protein families. As a consequence, the application of a standard matrix for the alignment of a determinate protein family could give inaccurate results, particularly if the amino acid composition differs from those used for the matrix construction. Still, only a few standard substitution matrices have been employed for database search and comparison of protein sequences during decades [19–21]. Nonetheless specific substitutions matrices for certain families of proteins are continuously developing [22–24]. These matrices, in many cases have proven to be more effective than the standard matrices in recognizing evolutionary relationships between the proteins of interest.

In this work, we computed a substitution matrix from a curated alignment of one thousand sequences of the TM regions of the GPCR rhodopsin family. Analysis of amino acid frequencies and values obtained from the matrix reveals patterns of residue replacements different from other standard substitution matrices. Charged and polar residues in particular seem to evolve at different rates than other amino acids. This observation could be related to the extraordinary diversification of the 7TM helical bundle in GPCRs for ligand recognition [25].

## Methods

### GPCR sequences retrieval and alignment

Class A GPCR protein sequences from the four main groups (α, β, δ and γ) and 13 sub-branches [5], including orphans, were obtained from the UniProt database from different biological sources [26]. This dataset was extended with the inclusion of 314 sequences from a curated set of functional human olfactory GPCR repertoire [27]. To avoid poorly aligned positions, UniProt and GPCRdb [14] annotations were used to identify TM segments and to remove the highly divergent intra and extracellular loops and the N- and C-terminal regions of the receptors. Boundaries of the TM helices were defined attending to the available crystal structures of class A GPCRs [28, 29]. Sequences corresponding to TMs 1–7 were aligned using the Win32 version of ClustalW 2.1 [30] and the closely related (>90 % identity) were excluded from the analysis. The resulting alignment was manually curated in order to achieve the optimal match between conserved sequence motifs present in the rhodopsin family [31] and small gaps were inserted in the TM2 and 5 according to previous studies [32]. This resulted in a final alignment of 1019 non-redundant TM GPCR sequences (see Additional file 1).

### Construction of GPCRtm

The alignment of the TM regions was used to generate a substitution matrix representing changes on GPCR sequences using an implementation of the methodology described by Henikoff *et al.* [20]. In this regard, the corresponding TM segments (1-7), which consist of multiple alignments of short regions (<40 amino acids), were treated as sequence blocks. As initial step, a transition count (frequency) table was computed to determine the total number of amino acid transitions pairs from each column of the alignment. After the transition count table was completed, observed and expected probability of transition were computed for each pair. The observed probability (*O*) for the amino acid pair (*i,j*) is the total number of transitions observed (from the frequency table) divided by the total number of transitions for the entire alignment.

$$ {O}_{ij}={f}_{ij}/{\displaystyle \sum_{i=1}^{20}}{\displaystyle \sum_{j=1}^i}{f}_{ij} $$

The expected probability (*e*) of occurrence for each (*i,j*) pair was calculated from the observed probabilities for the pair.

For a single residue:

$$ {p}_i={O}_{ii}+{\displaystyle \sum_{i\ne j}}\raisebox{1ex}{${O}_{ij}$}\!\left/ \!\raisebox{-1ex}{$2$}\right. $$

for an (*i,j*) pair:

$$ {e}_{ij}={p}_i{p}_j+{p}_j{p}_i=2{p}_i{p}_j\kern1.25em \mathrm{f}\mathrm{o}\mathrm{r}\kern0.5em i\ne j $$

when *i = j,*

$$ {e}_{ij}={p}_i{p}_j={p}_i^2 $$

Using the expected (*e*) and observed (*O*) probabilities of transitions, the substitution values were calculated from the odds ratio matrix, as the logarithm of odds, where each entry is obtained according to:

$$ {S}_{ij} = 2{ \log}_2\left({O}_{ij}/{e}_{ij}\right) $$

**Fig. 1:**
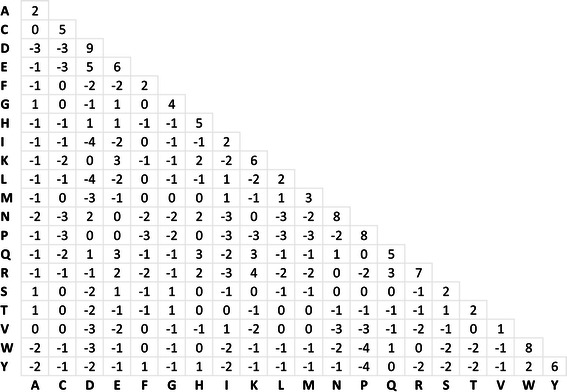
The G protein-coupled receptor transmembrane substitution matrix (GPCRtm)

The scaling factor of 2 is taken from *Henikoff* et al. [20] in order to facilitate comparisons. In the final 20 × 20 amino acid matrix (Fig. 1), substitutions values where rounded to the nearest integer value. In addition, we calculate the average mutual information per amino acid pair or relative entropy (*H*) according to:

$$ H={\displaystyle {\sum}_{i=1}^{20}{\displaystyle {\sum}_{j=1}^i{O}_{ij} \times {S}_{ij}}} $$

### Database searching and pairwise alignments

One hundred random sequences from different GPCR subfamilies, including the four main groups α, β, δ and γ [5], were used as queries in BLASTP searches executed with the AB-BLAST software (http://blast.advbiocomp.com/) against the pdbaa database (ftp://ftp.ncbi.nlm.nih.gov/blast/db/). Parameters to the customized gapped alignment score system for the GPCRtm were computed with the ALP program [33] (see Additional file 2). All BLASTP results were conducted with a gap existence = 15 and a gap extension = 2 scoring parameters, except for the BLOSUM62 matrix (gap existence = 11 and a gap extension = 1, default parameters). Matched comparisons of GPCRtm against JTTtm, PHAT, BLOSUM62 and BLOSUM45 matrices were calculated with the IBM SPSS Statistics for Macintosh, Version 22.0 using the exact McNemar 2-tailed tests (*p*-values). Pairwise sequence alignments were generated with the MAFFT (L-INS-i) software using default parameters [34, 35].

## Results and Discussion

### Amino acid compositional bias in the rhodopsin family of GPCRs

The average amino acid composition of the TM regions of the rhodopsin family was compared with amino acid frequencies derived from other studies (Table 1). As expected, the fraction of hydrophobic residues in the membrane spanning regions of GPCRs is similar to other TM proteins (JTTtm and PHDhtm) and is higher than in general proteins (BLOSUM62, and Swiss-Prot). Leucine is the most common occurring residue followed by valine and isoleucine. Nonetheless, there are differences in the amino acid composition of GPCRs. This is the case for charged and polar residues, with the exception of serine and threonine that behave similar in all datasets. The accumulated percentage for the R, K, H, D, E, N, and Q amino acids in the GPCRtm dataset (19.6 %) is in between JTTtm (9.5 %) and PHDhtm (9.9 %) datasets and BLOSUM62 (32.3 %) and Swiss-Prot (33.8 %) datasets. In addition, TM regions of the rhodopsin family are also characterized for a lower frequency of glycine (4.6 %) and a higher frequency of cysteine (3.6 %) residues relative to the other datasets. Given such differences in amino acid composition, we presume that general protein matrices such as the BLOSUM series and TM-derived protein matrices may not perform accurately in the alignment of the TM regions of GPCRs.

**Table 1:** Amino acid composition of substitution matrices and the Swiss-Prot database (%)

Amino acid	GPCRtm	JTTtm [44]	PHDhtm [22]	BLOSUM62 [20]	Swiss-Prot [45]
Ala (A)	8.0	10.5	8.8	7.4	8.3
Cys (C)	3.6	2.2	2.6	2.5	1.4
Asp (D)	2.1	0.9	1.4	5.4	5.5
Glu (E)	1.9	1.0	1.0	5.4	6.7
Phe (F)	7.3	7.7	9.3	4.7	3.9
Gly (G)	4.6	7.6	5.7	7.4	7.0
His (H)	2.1	1.7	1.1	2.6	2.3
Ile (I)	8.1	11.9	11.0	6.8	5.9
Lys (K)	3.4	1.1	0.9	5.8	5.8
Leu (L)	14.1	16.3	16.0	9.9	9.7
Met (M)	3.1	3.3	4.1	2.8	2.4
Asn (N)	3.4	1.8	2.2	4.5	4.1
Pro (P)	3.8	2.6	3.2	3.9	4.7
Gln (Q)	2.2	1.4	1.2	3.4	3.9
Arg (R)	4.5	1.6	2.1	5.2	5.5
Ser (S)	6.8	5.7	6.5	5.7	6.6
Thr (T)	5.6	5.2	5.3	5.1	5.3
Val (V)	9.2	11.9	11.0	7.3	6.9
Trp (W)	1.9	2.2	1.9	1.3	1.1
Tyr (Y)	4.3	3.2	4.7	3.2	2.9

### GPCRtm: a substitution matrix for the transmembrane regions of GPCRs

A curated alignment of more than one thousand membrane spanning sequences of class A GPCRs from different organisms were used for the generation of an amino acid substitution matrix (Fig. 1). The matrix was built using an approach similar to the one employed for the construction of the BLOSUM series of matrices [20]. Unlike BLOSUM matrices, built from sequence blocks of a variety of biological sources, we employ sequences of only GPCRs that accounts for the compositional bias in this family of receptors. Inspecting the diagonal elements of the matrix in the Fig. 1 we can estimate the mutability potential of each residue. Hydrophobic residues (V, L, I, A, F) display the highest level of relative mutability (corresponding to low values on the matrix, ≤ 2), whereas charged and polar residues are in general less mutable. Polar serine and threonine residues are special cases, displaying similar values than hydrophobic residues. These two amino acids, unlike other polar or charged residues, do not destabilize TM helices, as their hydrogen bonding potential can be satisfied by interacting with the carbonyl oxygen in the preceding turn of the same helix [36]. In contrast, N, D, R, W and P amino acids display the lowest level of relative mutability (corresponding to high values on the matrix, ≥ 7). All these residues display a high conservation pattern in at least one of TM helices of class A GPCRs [31, 37]: N in TM 1 (present in 98 % of the sequences), D in TM 2 (93 %), R in TM 3 (95 %), W in TM 4 (96 %) and P in TMs 5 (76 %), 6 (98 %) and 7 (93 %). Significantly, the position of these highly conserved amino acids in each helix is the same in the superimposition of the currently available crystal structures [38]. Positively (K, R, and H) and negatively (D, E) charged residues are easily interchangeable with each other. This could be due to a selection pressure to adapt the binding cavity of the TM bundle to the different chemical features of the ligands that, in many cases, display strong electrostatic properties (discussed below).

### Functional similarities of amino acids in GPCRtm. Comparison with other matrices

**Fig. 2:**
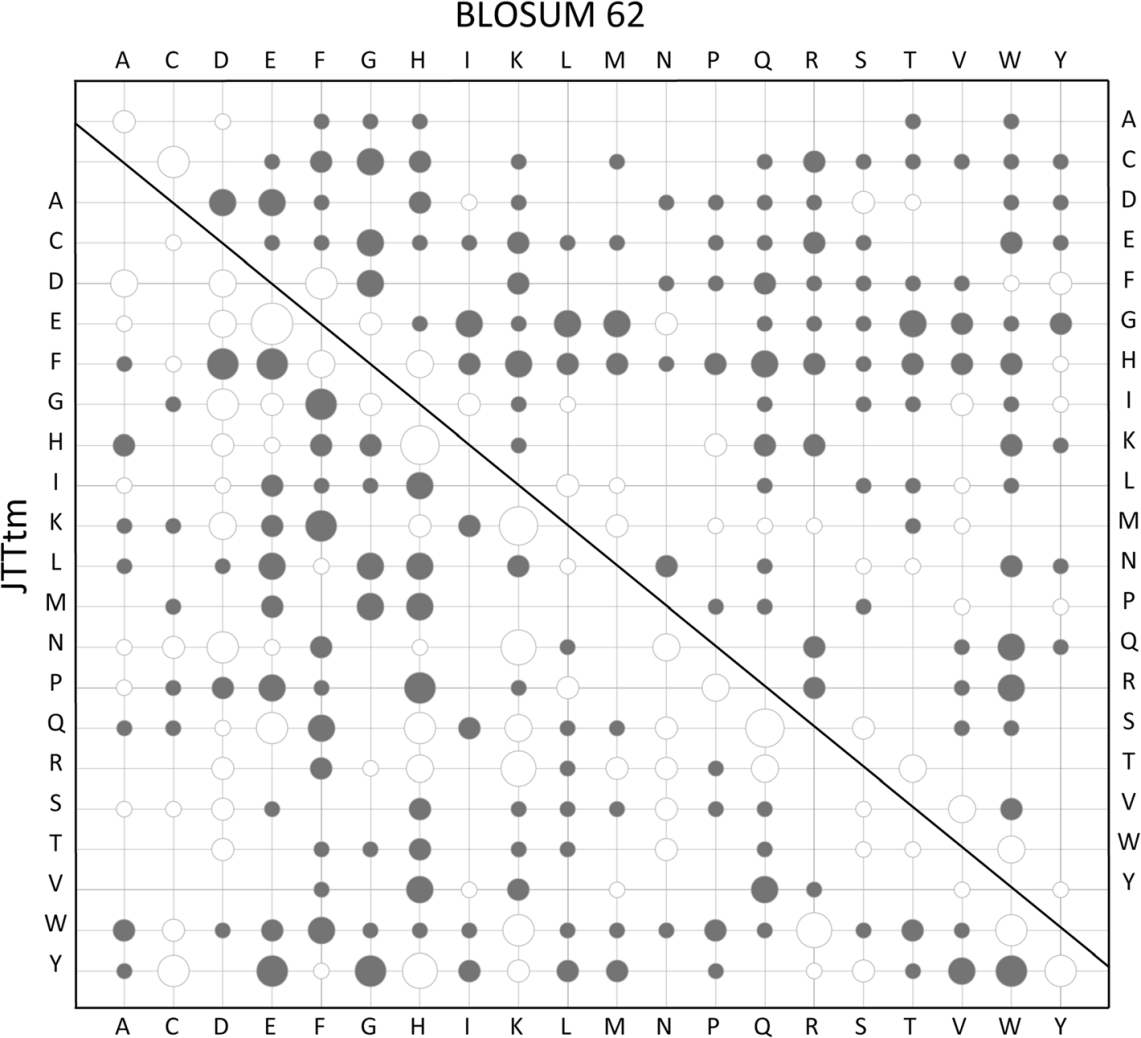
Bubble chart of the difference matrix obtained by subtracting from GPCRtm the JTTtm (*lower*) and BLOSUM62 (*upper*) substitution matrices. Positive and negatives values are showed in grey and white circles respectively. Bubbles are scaled according to the absolute value of the difference (numerical values are available in the supporting data)

GPCRtm (relative entropy, *H* = 0.6540) displays intermediate properties between matrices derived from general TM data sets (JTTtm, *H* = 0.5599 and PHAT, *H* = 0.5550) and for water-soluble globular proteins (BLOSUM62, *H* = 0.6979). A comparison of GPCRtm with other matrices is shown in Fig. 2 (see Additional file 3). In GPCRtm, charged and polar amino acids (K, R, H, D, E, N and Q) interchange with higher frequencies than in BLOSUM62 and lower than in JTTtm. In general, there is an intermediate performance of GPCRtm between general TM-derived and globular protein matrices with regard to the majority of charged and polar residues, which suggest a distinctive role of these amino acids in GPCRs.

**Fig. 3:**
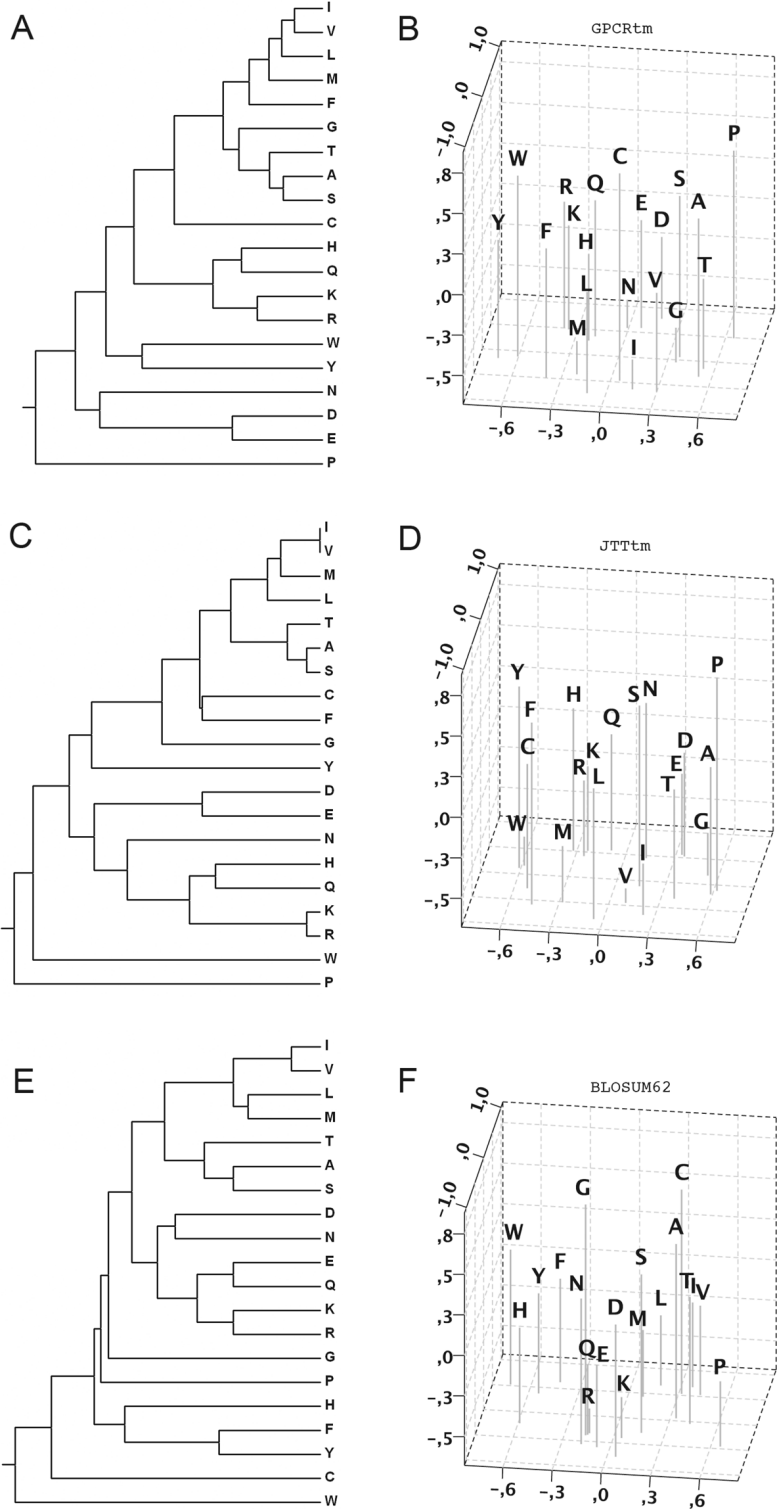
Unweight pair groups mean analysis dendograms (left) and multi-dimensional scaling projections (right) of the GPCRtm **a**, **b**; the JTTtm **c**, **d** and the BLOSUM62 **e**, **f** substitution matrices

One of the most important aspects of substitution matrices is amino acid grouping based on their chemical properties. These similarities could be easily visualized through the construction of dendograms and multi-dimensional projections to account for the correspondence of amino acids in the matrix (Fig. 3). Clearly, clustering of residues in GPCRtm, JTTtm and BLOSUM62 follow similar patterns, but with significant differences. The cluster of hydrophobic residues (I, V, L, M) is closer to the cluster of small amino acids (A, S, T) in all cases. However, GPCRtm differs from other matrices in that phenylalanine is grouped with hydrophobic amino acids (the I, V, L, M, F cluster), whereas in BLOSUM62 is grouped with the aromatic tyrosine and in JTTtm with cysteine. Similarly, glycine is clustered together with the other small amino acids (A, S, T), in contrast to other matrices in which is grouped alone. Histidine clusters with positively charged and polar amino acids in GPCRtm and JTTtm, in contrast to BLOSUM62. This residue is grouped with glutamine in GPCRtm and JTTtm, probably due to its hydrogen bond donor/acceptor properties, whereas in BLOSUM62 is grouped with phenylalanine and tyrosine probably due to its aromaticity. GPCRtm clusters tryptophan and tyrosine together, preserving aromaticity and hydrogen bond capacity, whereas in the other matrices tryptophan is unaccompanied. The negatively charged aspartate and glutamate form one group in GPCRtm and JTTtm, while in BLOSUM62 aspartate pairs with asparagine and glutamate with glutamine. In this regard, positive (K, R) and negative (D, E) residues are grouped at closer distance in BLOSUM62. In contrast, positive and negative residues are distant in GPCRtm and JTTtm. Interestingly, the distance between branches containing opposite charged residues in GPCRtm is larger than in JTTtm, suggesting than the sign of the charge is apparently more conserved in the GPCR TM sequences than in a general set of TM proteins.

**Fig. 4:**
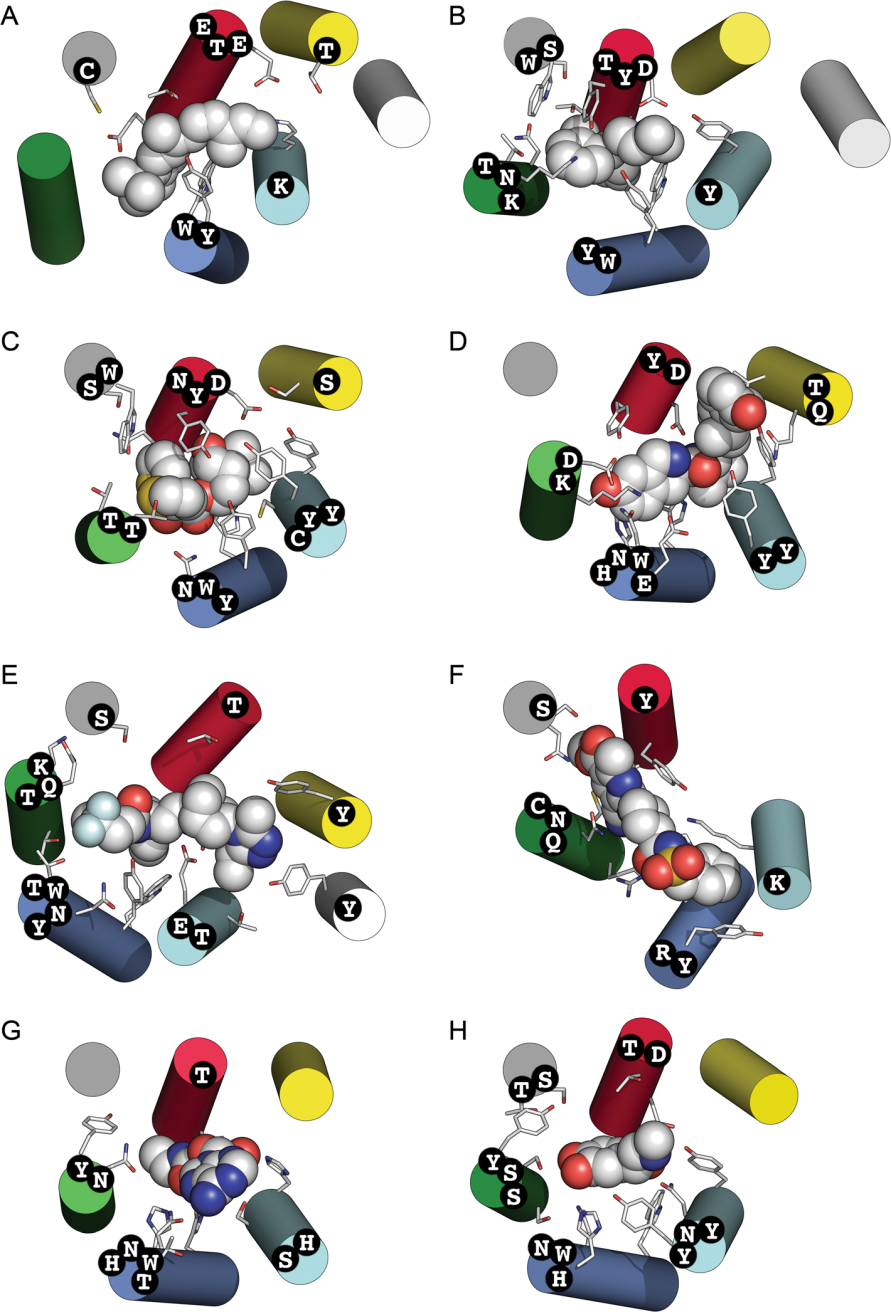
Diversity of ligand binding interactions involved polar and charge residues in the TM region of the rhodopsin family of GPCRs. The crystal structures corresponding to: **a** Rhodopsin (PDBid: 1U19), **b** Histamine H_1_R (3RZE), **c** Muscarinic M_3_R (4DAJ), **d** Opioid κ-OR (4DJH), **e** Chemokine CCR_5_ (4MBS), **f** Purinergic P_2_Y_12_R (4NTJ), **g** Adenosine A_2_AR (2YDV) and **h** Adrenergic β_2_AR (4LDO). Polar and charged residues of the receptors at 4 Å distance of ligands (*in vdW spheres*) are displayed as sticks and named in the corresponding helices (circular labels). The color code of the helices is: TM1 (*light grey*), TM2 (*yellow*), TM3 (*red*), TM4 (*grey*), TM5 (*green*), TM6 (*darkblue*) and TM7 (c*yan*). All structures are oriented with the TM4 perpendicular to the plane

Overall, the results show that GPCRtm prioritized the reactivity properties of the amino acids over their bulkiness. In this way, hydrophobic residues (including phenylalanine), which are key in TM regions, are clustered together. On the other side, the hydrogen bond capacity and electronic properties of the amino acids tend to be maintained in GPCR sequences. Thus, the H/Q, K/R, E/D/N and W/Y pairs together. These residues contribute largely to the diversity of interactions between ligands and the 7TM bundle as can be observed in the 3D structures of ligand-receptor complexes in some members of the rhodopsin family (see Fig. 4). In this respect, GPCRs are distinguished from most TM proteins for their ability to interact with a diverse variety of chemical entities.

### Evaluation of the GPCRtm matrix

The GPCRtm matrix was tested on sequence similarity searches and pairwise alignments. The results of GPCRtm were compared with commonly used amino acid exchange matrices, the JTTtm and PHAT transmembrane matrices and the general-purpose BLOSUM45 and BLOSUM62 matrices. At high sequence identity values (above the twilight zone) all matrices behave similarly. However, as sequence identity falls below 40 %, significant differences emerged. Table 2 shows a comparison among the different substitution models in BLASTP database searches for one hundred GPCR queries against the PDB database [39]. As observed in the table, the GPCRtm matrix performs better than other matrices. The second best performance was achieved by the closely related PHAT matrix, followed by the BLOSUM62, BLOSUM45 and JTTtm matrices, respectively.

**Table 2:** Comparative analysis of the GPCRtm performance regarding general-purpose substitution matrices in BLASTP searches of one hundred GPCR protein queries against the PDB database

Test matrix	No. of queries GPCRtm better	No. of queries GPCRtm worst	No. of queries GPCRtm the same	*p*-value
JTTtm	21	0	79	<0.001**
PHAT	8	0	92	0.008*
BLOSUM62	9	1	90	0.021*
BLOSUM45	12	1	87	0.003*

*p-*values were calculated by McNemar’s test (* significant differences at α = 0.05, ** significant differences at α = 0.001)

Criteria for the performance evaluation were based on the recognition of the closest homologue with known three-dimensional structure for a determinate query, according to the well-established GPCR classification systems [4, 5]. Table 3 illustrates an example for the adrenergic receptor (ADR) subfamily of GPCRs. ADRs interact with the endogenous catecholamines adrenaline and noradrenaline and constitute essential regulators of central and peripheral metabolic functions [40]. These receptors are classified into three main groups: the α_1_-, α_2_- and β-adrenoceptors. Only two members (β _1_- or ADRB1 and β_2_- or ADRB2) have been solved by X-Ray crystallography, constituting the reference structures for the adrenoceptors subfamily [41]. According to the results shown in Table 3, the GPCRtm matrix performs better than general-purpose matrices in BLASTP searches, resolving a receptor of the same subfamily (ADRB1 or ADRB2) as a first hit for searches involved the nine ADR subtypes as queries. On the other hand, in some instances (at lower identities) the standard matrices deliver as best hit a receptor of a different GPCR subfamily.

**Table 3:** Results of BLASTP database searches using the nine human adrenergic receptor subtypes as queries against the Protein Data Bank. The table displays only the first hit (lower E-value) of each search (IUPAC name of the receptor and PDBid code in parenthesis) followed by the sequence identity values in the aligned regions and the corresponding bit scores for the GPCRtm and general substitution matrices

	GPCRtm				JTT				PHAT				BLOSUM62				BLOSUM45			
Query Receptor	First Hit	Id. (%)	Score (bits)	E-value	First Hit	Id. (%)	Score (bits)	E-value	First Hit	Id. (%)	Score (bits)	E-value	First Hit	Id. (%)	Score (bits)	E-value	First Hit	Id. (%)	Score (bits)	E-value
ADA1A	ADRB1 (2VT4)	34	140.9	1.0e^−55^	ACM2 (4MQS)	30	140.0	2.3e^−55^	ADRB2 (3KJ6)	36	226.3	8.8e^−62^	5HT1B (4IAQ)	35	133.9	7.5e^−57^	5HT1B (4IAQ)	35	140.9	5.0e^−59^
ADA1B	ADRB1 (2VT4)	35	139.4	3.5e^−56^	ADRB1 (2VT4)	34	133.9	4.8e^−59^	ADRB2 (3KJ6)	34	215.3	3.5e^−58^	ADRB1 (2Y00)	35	139.3	1.1e^−55^	ADRB1 (2Y00)	35	141.2	4.6e^−56^
ADA1D	ADRB1 (2VT4)	35	154.3	3.3e^−58^	ADRB1 (2VT4)	35	133.9	4.8e^−56^	ADRB1 (2VT4)	36	156.6	1.6e^−60^	ADRB1 (2VT4)	36	150.4	1.0e^−57^	ADRB1 (2VT4)	36	150.3	9.1e^−58^
ADA2A	ADRB1 (2VT4)	40	130.4	6.0e^−50^	ACM2 (4MQS)	26	126.8	4.2e^−49^	ADRB2 (3D4S)	29	163.5	8.8e^−53^	5HT1B (4IAR)	39	144.3	5.3e^−56^	5HT1B (4IAQ)	41	147.0	1.0e^−57^
ADA2B	ADRB2 (2R4S)	30	128.9	8.1e^−47^	DRD3 (3PBL)	30	195.8	4.5e^−53^	DRD3 (3PBL)	31	215.9	1.3e^−56^	5HT1B (4IAR)	36	135.4	8.0e^−55^	5HT1B (4IAR)	36	142.9	2.1e^−58^
ADA2C	ADRB1 (2VT4)	35	118.5	1.2e^−49^	ADRB1 (2VT4)	34	108.1	5.1e^−49^	ADRB1 (2VT4)	37	130.2	2.8e^−53^	5HT1B (4IAR)	35	134.7	6.8e^−56^	5HT1B (4IAR)	34	139.4	1.7e^−58^
ADRB1	ADRB1 (2Y00)	77	308.0	2.6e^−135^	ADRB1 (3KJ6)	57	241.1	1.5e^−99^	ADRB1 (2Y00)	77	338.1	1.1e^−148^	ADRB1 (2Y00)	77	317.4	2.5e^−130^	ADRB1 (2Y00)	77	319.6	2.9e^−132^
ADRB2	ADRB2 (2R4R)	99	696.1	5.1e^−204^	ADRB2 (2R4R)	99	624.7	3.4e^−182^	ADRB2 (2R4R)	99	791.0	2.3e^−232^	ADRB2 (2R4R)	99	686.4	1.2e^−200^	ADRB2 (2R4R)	99	678.6	2.1e^−198^
ADRB3	ADRB1 (2Y00)	53	201.6	2.3e^−86^	ADRB1 (2Y00)	53	185.2	3.7e^−78^	ADRB1 (2Y00)	56	220.0	2.4e^−95^	ADRB1 (2Y00)	56	215.7	6.6e^−89^	ADRB1 (2Y00)	56	217.6	2.4e^−90^

**Fig. 5:**
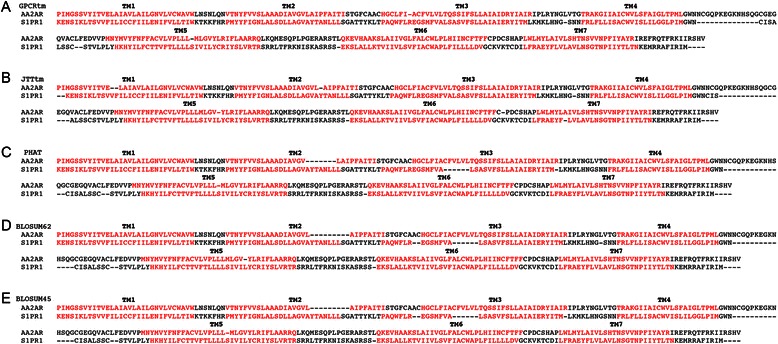
Example of pairwise alignments of the adenosine AA2AR and sphingosine-1-phosphate S1PR1 amino acid sequences using: GPCRtm (**a**), JTTtm (**b**), PHAT (**c**), BLOSUM62 (**d**) and BLOSUM45 (**e**) substitution matrices. Transmembrane regions TM 1 to 7 appear outlined in red according on the crystallographic 3D structural data for each receptor (PDBid: 3EML and 3V2Y). Pairwise sequence alignments were done with MAFFT program [35]

One of the best ways to test alignment accuracies is to compare the results with structure-based information derived from three-dimensional structural data. In this regard, the GPCRmt matrix was tested on pairwise sequence alignments of class A GPCR whose structures are known. Figure 5 shows the result of the alignment between the adenosine A_2A_ receptor (AA2AR) and sphingosine-1-phosphate receptor 1 (S1PR1) using different substitution matrices. Both receptors are members of the MECA receptor cluster of the rhodopsin family [5] with known three-dimensional structures [42, 43]. In this example, the resulting alignments denote the accuracy of the GPCRtm to correctly align the TM helices of both receptors, whereas generalized matrices fails to correctly align some of the TM regions. According to these results, the GPCRtm matrix improve the detection of closest homologues and produce accurate alignments in the TM regions of GPCRs, even at low sequence identities. This is particularly relevant in the development of homology models for structure-based drug discovery, which in many cases are generated from low sequence identity alignments due to the limited number of GPCRs crystallographic structural templates [32].

## Conclusions

We present GPCRtm, an amino acid substitution matrix for the TM regions of the rhodopsin family of GPCRs. GPCRtm is evolutionary consistent with amino acid frequencies and actual changes occurring within this protein family. Analysis of the matrix reveals the differences between GPCRs and other membrane proteins and proteins in general. This is evidenced by distinctive frequencies of polar and charged residues and a prevalence of reactivity over size in the contribution of the conservation pattern. These observations stresses the relatively high importance of charged and polar amino acids in this family of receptors with regard to other membrane proteins, possibly due to their versatility in ligand interaction. In this regard, this matrix could assist in evolutionary studies, improving the classification and increasing the accuracy of phylogenetic reconstruction for members of this family of membrane receptors. The GPCRtm, besides important from a theoretical point of view, could be used in sequence alignments and database searches of class A GPCRs.

### Availability of supporting data

The data sets supporting the results of this article are included within the article and its additional files.

## Additional files

Additional file 1:
**Compilation of subfamilies, principal clades and sequence alignment of class A GPCR transmembrane regions (TM1 to 7) used to generate the GPCRtm substitution matrix.**

Additional file 2:
**Gumbel distribution statistical parameters λ and κ and the relative entropy H for gapped local alignment scores calculated for the GPCRtm matrix operating at different gap penalties.**

Additional file 3:
**Difference matrix obtained by subtracting from the GPCRtm the JTTtm and the BLOSUM62 substitution matrices.**

## Abbreviations

GPCR: G Protein-Coupled Receptor
TM: Transmembrane
GPCRtm: G Protein-Coupled Receptor transmembrane substitution matrix
BLOSUM: Blocks Substitution Matrix
JTTtm: Jones, Taylor and Thornton mutation data matrix for transmembrane proteins
