# Supplementary material for: GPCRtm: An amino acid substitution matrix for the transmembrane region of class A G Protein-Coupled Receptors
Source: BMC Bioinformatics. 2015 Jul 2;16:206. doi: 10.1186/s12859-015-0639-4 (PMC4489126; doi:10.1186/s12859-015-0639-4)
Supplement: Additional file 2: — Gumbel distribution statistical parameters λ and κ and the relative entropy H for gapped local alignment scores calculated for the GPCRtm matrix operating at different gap penalties. [file 12859_2015_639_MOESM2_ESM.docx]

Gumbel distribution statistical parameters λ and κ and the relative entropy *H* for gapped local alignment scores calculated using the ALP program [34] for the GPCRtm matrix operating at different gap penalties parameters.

| Gap penalty parameters  (open= Q, extension= R) | λ | κ | *H* |
| --- | --- | --- | --- |
| Q=9 R=2 | 0.3218 | 0.0506 | 0.2013 |
| Q=10 R=4 | 0.3420 | 0.0749 | 0.2470 |
| Q=11 R=1 | 0.3121 | 0.0383 | 0.1819 |
| Q=15 R=2 | 0.3448 | 0.0846 | 0.2542 |
|  |  |  |  |
