# Supplementary material for: GPCRtm: An amino acid substitution matrix for the transmembrane region of class A G Protein-Coupled Receptors
Source: BMC Bioinformatics. 2015 Jul 2;16:206. doi: 10.1186/s12859-015-0639-4 (PMC4489126; doi:10.1186/s12859-015-0639-4)
Supplement: Additional file 3: — Difference matrix obtained by subtracting from the GPCRtm the JTTtm and the BLOSUM62 substitution matrices. [file 12859_2015_639_MOESM3_ESM.docx]

Difference matrix obtained by subtracting from the GPCRtm the JTTtm (*Lower*) and the BLOSUM62 substitution matrices (*Upper*)

BLOSUM62

|  | **A** | **C** | **D** | **E** | **F** | **G** | **H** | **I** | **K** | **L** | **M** | **N** | **P** | **Q** | **R** | **S** | **T** | **V** | **W** | **Y** |  |
| --- | --- | --- | --- | --- | --- | --- | --- | --- | --- | --- | --- | --- | --- | --- | --- | --- | --- | --- | --- | --- | --- |
|  | -2 | 0 | -1 | 0 | 1 | 1 | 1 | 0 | 0 | 0 | 0 | 0 | 0 | 0 | 0 | 0 | 1 | 0 | 1 | 0 | **A** |
|  |  | -4 | 0 | 1 | 2 | 3 | 2 | 0 | 1 | 0 | 1 | 0 | 0 | 1 | 2 | 1 | 1 | 1 | 1 | 1 | **C** |
| **A** | 0 |  | 3 | 3 | 1 | 0 | 2 | -1 | 1 | 0 | 0 | 1 | 1 | 1 | 1 | -2 | -1 | 0 | 1 | 1 | **D** |
| **C** | 0 | -1 |  | 1 | 1 | 3 | 1 | 1 | 2 | 1 | 1 | 0 | 1 | 1 | 2 | 1 | 0 | 0 | 2 | 1 | **E** |
| **D** | -3 | 0 | -3 |  | -4 | 3 | 0 | 0 | 2 | 0 | 0 | 1 | 1 | 2 | 1 | 1 | 1 | 1 | -1 | -2 | **F** |
| **E** | -1 | 0 | -3 | -7 |  | -2 | 1 | 3 | 1 | 3 | 3 | -2 | 0 | 1 | 1 | 1 | 3 | 2 | 1 | 2 | **G** |
| **F** | 1 | -1 | 4 | 4 | -3 |  | -3 | 2 | 3 | 2 | 2 | 1 | 2 | 3 | 2 | 1 | 2 | 2 | 2 | -1 | **H** |
| **G** | 0 | 1 | -4 | -2 | 4 | -2 |  | -2 | 1 | -1 | 0 | 0 | 0 | 1 | 0 | 1 | 1 | -2 | 1 | -1 | **I** |
| **H** | 2 | 0 | -2 | -1 | 2 | 2 | -6 |  | 1 | 0 | 0 | 0 | -2 | 2 | 2 | 0 | 0 | 0 | 2 | 1 | **K** |
| **I** | -1 | 0 | -1 | 2 | 1 | 1 | 3 | 0 |  | -2 | -1 | 0 | 0 | 1 | 0 | 1 | 1 | -1 | 1 | 0 | **L** |
| **K** | 1 | 1 | -3 | 2 | 4 | 0 | -2 | 2 | -6 |  | -2 | 0 | -1 | -1 | -1 | 0 | 1 | -1 | 0 | 0 | **M** |
| **L** | 1 | 0 | 1 | 3 | -1 | 3 | 3 | 0 | 2 | -1 |  | 2 | 0 | 1 | 0 | -1 | -1 | 0 | 2 | 1 | **N** |
| **M** | 0 | 1 | 0 | 2 | 0 | 3 | 3 | 0 | 0 | 0 | 0 |  | 1 | 1 | 0 | 1 | 0 | -1 | 0 | -1 | **P** |
| **N** | -1 | -2 | -4 | -1 | 2 | 0 | -1 | 0 | -5 | 1 | 0 | -3 |  | 0 | 2 | 0 | 0 | 1 | 3 | 1 | **Q** |
| **P** | -1 | 1 | 2 | 3 | 1 | 0 | 4 | 0 | 1 | -2 | 0 | 0 | -3 |  | 2 | 0 | 0 | 1 | 3 | 0 | **R** |
| **Q** | 1 | 1 | -1 | -4 | 3 | 0 | -4 | 2 | -3 | 1 | 1 | -2 | 0 | -6 |  | -2 | 0 | 1 | 1 | 0 | **S** |
| **R** | 0 | 0 | -2 | 0 | 2 | -1 | -3 | 0 | -5 | 1 | -2 | -2 | 1 | -3 | 0 |  | -3 | 0 | 0 | 0 | **T** |
| **S** | -1 | -1 | -2 | 1 | 0 | 0 | 2 | 0 | 1 | 1 | 1 | -2 | 1 | 1 | 0 | -1 |  | -3 | 2 | 0 | **V** |
| **T** | 0 | 0 | -2 | 0 | 1 | 1 | 2 | 0 | 1 | 1 | 0 | -2 | 0 | 1 | 0 | -1 | -1 |  | -3 | 0 | **W** |
| **V** | 0 | 0 | 0 | 0 | 1 | 0 | 3 | -1 | 2 | 0 | -1 | 0 | 0 | 3 | 1 | 0 | 0 | -1 |  | -1 | **Y** |
| **W** | 2 | -2 | 1 | 2 | 3 | 1 | 1 | 1 | -4 | 1 | 1 | 1 | 2 | 1 | -5 | 1 | 2 | 1 | -4 |  |  |
| **Y** | 1 | -4 | 0 | 4 | -1 | 4 | -5 | 2 | -2 | 2 | 2 | 0 | 1 | 0 | -1 | -2 | 1 | 3 | 4 | -4 |  |
|  | **A** | **C** | **D** | **E** | **F** | **G** | **H** | **I** | **K** | **L** | **M** | **N** | **P** | **Q** | **R** | **S** | **T** | **V** | **W** | **Y** |  |

JTTtm
