# Supplementary material for: GPCRtm: An amino acid substitution matrix for the transmembrane region of class A G Protein-Coupled Receptors
Source: BMC Bioinformatics. 2015 Jul 2;16:206. doi: 10.1186/s12859-015-0639-4 (PMC4489126; doi:10.1186/s12859-015-0639-4)

GPCRs included in the alignment:

By subfamily:

Prostaglandin:	PD2R, PE2Rs, PF2R, PI2R, TA2R
Amine:	5THRs, ACMs, ADAs, ADRAs, ADRBs, DOPRs, DRDs, HRHs, TAARs
Opsin:	OP1Ss, OPNs, OPSs, RGR
MCH:	MCHRs
Melatonin:	MR1s, MTR1s
MECA:	MSHR, ACTHR, CNRs, LPARs, MCRs, S1PRs
Peptide (ß-branch):	BRS3, BRS4, CCKAR, EDNRs, ETBR2, GHSR, GNRHR, GNRR2, GRPR, NPRs, NPSR1, GASR, NKRs, NMBR, NUMRs, NPBWs, NPFFs, NPYRs, NTRs, OXRs, OXYR, VRs
SOG:	GALRs, KISSR, OPRs, SSRs
Chemokine:	AGTRs, APJs, BKRBs, C3AR, C5ARs, CCRs, CXCRs, CCRLs, CML1, CX3C1, CC1L1, CCBP2, XCR1, FPRs
MAS-related:	MAS, MAS1L, MRGAs, MRGBs, MRGRs, MRGXs
Glycoprotein:	FSHR, LGRs, LSHR, RL3Rs, RXFPs, TSHR
Purine:	AARs, CLTRs, LPARs, P2RYs, P2Ys, PARs, PTAFR
Olfactory receptors:	#ORs
Orphan receptors:	GPs, GPRs
GPCR not classified:	ANR, AR, AVT, CAPAR, CCAPR, CTRs, FMAR, FFARs, G109A, GLHR, GPBAR, GPER, GUTR1, ITR, MOODY, MTR, MTR1s, OARs, OCTBs, OGR1, OXER1, OXGR1, PKRs, PRLHR, PSYR, QRFPR, SUCR1, TLRs, TRFR, UR2R
Probable GPCR:	PSP24, TKR1, UL33, US27, US28, V021, V027, V206, VK02, YKR5, YMJC, YQH2, YWO1

By species:

Mammals	HUMAN: Human, MOUSE: Mouse, RAT: Rat, RATRT: Black rat, CHAPN: Desert pocket mouse, BOVIN: Cow, CALJA: Marmoset, CALPD: Opossum, CANFA: Dog, CAPHI: Goat, CAVPO: Guinea pig, CERPY: Vervet monkey, DELDE: Dolphin, FELCA: Cat, GALSE: Bushbaby, GORGO: Gorilla, HAPGR: Grey gentle lemur, HORSE: Horse, MACFA: Crab-eating macaque, MACMU: Rhesus macaque, MACEU: Wallaby, MAMPR: Mammoth, MANSP: Sphinx, MERUN: Jird, MESAU: Hamster, PONAB: Sumatran Orangutan, PONPY: Bornean Orangutan, PIG: Pig, PANTR: Chimpanzee, PANPA: Pygmy chimpazee, PAPHA Baboon, PYGAV: Langur, RABIT: Rabit, SHEEP: Sheep, SMICR: Dunnart, TRIVU: Possum, TUPMI: Shrew, VARVR: Red ruffed Lemur, VULVU: Fox
Fish	ANGAN: Anguilla, ASTFA: Astyanax, ATHBO: Smelt, BRABE: Lancelet, CLAGA: Catfish, CARAU: Goldfish, CATCO: Sucker, CONCO: Muraena, CHELB: Mullet, DANRE: Zebrafish, DICLA: Seabass, GADMO: Cod, GALML: Blackmouth Catshark, ICTPU: Channel catfish, LABOS: Wrasse, LITMO: Seabream, NEOSA: Squirrelfish, ONCMY: Trout, OREMO: Mozambique tilapia, ORENI: Nile tilapia, ORYLA: Killifish, POERE: Guppy, POMMI: Sand goby, RUTRU: Roach, SALSA: Salmon, SARPI: Pilchard, SCYCA: Smal-spotted catshark, TAKRU: Takifugu, TETFL: Pufferfish, ZEUFA: John Dory, ZOSOP: Goby
Amphibia	BOMOR: Oriental toad, BUFMA: Giant toad, BUFBU: European toad, XENLA: Clawed frog, XENTR: Silurana
Bird	CHICK: Chicken, COLLI: Rock dove, MELUD: Budgerigar, MELGA: Turkey
Reptile	ANOCA: Chameleon, ALLMI: Alligator, GECGE: Gekko, PODSI: Lizard
Crustacean	HEMSA: Crab, PROCL: Crayfish
Molluscs	APLCA: Sea hare, ENTDO: Giant octopus, LOLFO: Squid, LYMST: Pond snail, OCTVU: Octopus, PATYE: Scallop
Insects	APIME: bee, BOMMO: Silkworm, CALVI: Blowfly, CATBO: Ant, DROME: Fruit fly, DROVI: Fruit fly, HELVI: Budworm, LOCMI: Migratory locust, MANSE: Hornworm, SCHGR: Desert locust, SPHSP: Mantis
Worms	CAEEL: Roundworm, EISFO: Dung-worm
Plants	ANTEL: Anemone, BALAM: Lophiarella
Viruses	HHV6U: Herpesvirus 6A, MCMVS: Mosaic virus, HCMVA: Herpesvirus 5, HCMVT: Herpesvirus 5, FOWPV: Fowlpox virus, SWPVK: Swinepox virus
Others	LIMPO: Horseshoe crab, PETMA: Lamprey

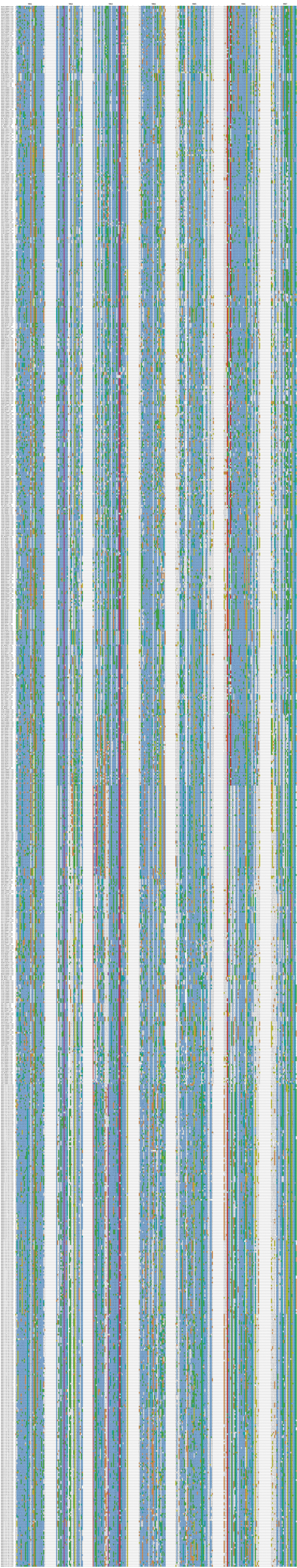

Supplement: Additional file 1: — Compilation of subfamilies, principal clades and sequence alignment of class A GPCR transmembrane regions (TM1 to 7) used to generate the GPCRtm substitution matrix. [file 12859_2015_639_MOESM1_ESM.pdf]
